# Changes in Movement Coordination Associated With Skill Acquisition in Baseball Batting: Freezing/Freeing Degrees of Freedom and Functional Variability

**DOI:** 10.3389/fpsyg.2020.01295

**Published:** 2020-06-25

**Authors:** Rob Gray

**Affiliations:** Human Systems Engineering, Arizona State University, Mesa, AZ, United States

**Keywords:** skill acquisition, baseball, perception-action, Bernstein, motor control

## Abstract

How do baseball batters solve the problem of coordinating the timing of the different phases of movement to generate a powerful swing that is appropriately adjusted for the trajectory of the pitch? How does the development of this coordination solution depend on the structure of practice? Previously unpublished ground reaction force (GRF) data were analyzed to investigate the swing coordination changes that were associated with the changes in batting performance found in the training study by Gray (2017). From pre–post training, there were significant increases in the magnitude of correlations between adjacent swing phases, significant increases in good variability (changes that keep the swing within the required temporal constraint), significant decreases in bad variability (changes that move the swing outside the temporal constraint), and stronger evidence of online adjustments of the different swing phases. These effects were significantly larger for the virtual environment (VE) Adaptive group from the Gray (2017) study that had higher variability in practice conditions. Across all participants, there were significant correlations between the changes in good and bad variability from pre–post training and measures of batting from VE and real hitting tests, and statistics from league play. These findings suggest that baseball batters solve the problem of coordination by developing functional variability and coupling between swing phases (Katsumata, 2007), which can be facilitated by having more variability in practice conditions.

## Introduction

The question of how the movement of our different body parts becomes coordinated when acquiring a new perceptual-motor skill has long been of interest. For example, when learning to hit a baseball, how should my lower body be moved with respect to my upper body? Bernstein (1967) captured the challenge involved in movement coordination in his now well-known degrees of freedom problem. He observed that for any motor skill, there are multiple, redundant degrees of freedom. In other words, there are a very large number of possible movement solutions that can be used to achieve the same outcome. For example, to a hit a baseball, I could move the bat via a rotation of my hands around the wrist joint, a rotation of my lower arms around the elbow joint, a rotation of my arms around the shoulder joint, a rotation of my upper body around the hip joint, or any combination of these movements. Furthermore, each of these joints can be rotated in different ways (e.g., there are six different muscles that move the elbow). Each of these rotations (and the associated muscle commands involved) is a “degree of freedom” in movement coordination as it represents a possible way to achieve the performer’s goal. The problem for Bernstein was: how does our perceptual-motor system determine the combination of movements to use when we are given so many options? Thus, he defined coordination as “the process of mastering redundant degrees of freedom of the moving body, in other words its conversion to a controllable system.”

Bernstein (1967) proposed that how we solve this degrees of freedom problem and achieve coordination depends on the stage of learning. When we are first learning a new skill, he proposed that the most effective solution will involve “freezing” degrees of freedom. Freezing essentially involves reducing the number of potential movement solutions available (in other words, reducing the number of degrees of freedom) to simplify the choice problem. There are two ways Bernstein proposed that this would occur. The first involves rigidly fixing separate degrees of freedom by not using particular joints or muscles during movement. For example, a person learning to swing a bat might choose to lock their wrist, and not change its angle during movement. Obviously, if a body part is held rigid and fixed, it is no longer a degree of freedom in movement so the total number of potential solutions has been reduced.

The second type of freezing proposed by Bernstein was introducing strong, temporary couplings between degrees of freedom. Instead of just keeping a particular body part rigid and locked, I can couple its movement to another body part, so they move together, in phase. For example, when swinging a bat, I could extend my wrist and elbow at the exact same rate and at the same time. By coupling body parts, the degrees of freedom are cut in half: instead of having to control the movement of two things, you just make one choice that covers two joint movements. Bernstein proposed that freezing would occur in a distal to proximal direction; that is, body parts in the periphery (that were further from the center of the body) were the ones that were frozen first, followed by ones that were closer (or more proximal), if necessary. However, while freezing simplifies the problem of movement coordination, a frozen movement solution will typically not be an optimal one and it is likely to be very inflexible, inefficient, and not very powerful.

Later in the learning process, once the performer has achieved some mastery over their skill, Bernstein (1967) proposed that more efficient solutions to the degrees of freedom problem would be used. Specifically, he proposed that further development of the skill would involve the gradual unfreezing or “freeing” of degrees of freedom. Again, this involved two aspects. First, the body parts that were rigidly fixed would now be free to move to allow for more flexibility. Bernstein again proposed that this would occur in a systematic order: proximal to distal, cephalocaudal, and ulnar to radial.

The second aspect of this freeing state proposed by Bernstein was the development of functional coupling between degrees of freedom (or body parts), what he referred to as motor synergies. For example, when solving the problem of what to do with my wrist and elbow when swinging a bat, I could have the rotations of the two joints work together and compensate for each other. If the movement of the bat caused by the rotation around the elbow joint is too slow to get the bat to the ball in time, I could increase the rate of rotation about my wrist joint and vice versa. For Bernstein, the development of motor synergies represents a superior solution to the degrees of freedom problem as compared to freezing because it allows the performer to be more adaptable to the ever-changing internal state of the body and the external environment (what he termed “context-conditioned variability”).

But how do movement synergies actually simplify the problem of movement coordination? For example, in hitting, if I go from freezing my wrist joint to coupling it with my elbow joint, haven’t I just increased the number of degrees of freedom again? To address this issue, Tuller and Turvey (1982) introduced the concept of a coordinative structure in motor control, which is a linkage between body segments such that they are constrained to act as one functional unit. Coordinative structures simplify the complexity of movement (and help to solve the degrees of freedom problem) by writing an equation of constraint which applies to a set of muscles and joints, thus treating them as a single unit. In essence, this creates an autonomous, self-regulatory mechanism. As long as the constraint is met, the system organizes itself within. So, in the baseball batting example, the movement of my wrist and elbow could be controlled using the equation of constraint: the combined rotation must get the bat to the hitting zone at a time equal to the time of arrival of the ball. In this view, motor learning is discovering the right kinds of constraint over separate parts of the body to achieve the goal action.

### Previous Research on Solving the Degrees of Freedom Problem

What evidence is there that movement coordination when learning a complex perceptual-motor skill involves the processes of freezing and freeing proposed by Bernstein? Surprisingly, there have been a relatively small number of studies that have evaluated his seminal ideas. In a recent systematic review, Guimarães et al. (2020) identified only 13 studies that have investigated freezing and freeing of degrees of freedom in motor learning. It was found that 10/13 studies provided evidence consistent with Bernstein’s hypothesis that freezing degrees of freedom is a strategy used early in learning.

Freezing has been assessed in previous research using two primary dependent measures: the joint range of motion (JROM) and the cross-correlation between the motion of joints. It has been proposed that JROM is directly related to Bernstein’s freezing–freeing dimension (Vereijken et al., 1992). In other words, we should expect to see lower values of JROM early in learning (consistent with Bernstein’s first type of freezing) followed by larger values after the performer has practiced the task for an extended period (consistent with freeing). This pattern of low to high JROM across practice sessions has been found in studies of motor learning tasks including simulated slalom skiing (Vereijken et al., 1992), kicking a soccer ball (Anderson and Sidaway, 1994; Hodges et al., 2005; Chow et al., 2008), dart throwing (Didier et al., 2013), and serving a racquetball (Smith et al., 2001). It should be noted, however, that this increase in JROM only occurs for select joints involved in the motion (not all of them) and does not necessarily follow a unidirectional, linear pattern with learning. For example, in their 7-day training study of chipping a soccer ball over a barrier, Hodges et al. (2005) found that JROM in the hip decreased from day 1 to day 5 and then started to increase. A reverse pattern was observed in the knee and ankle, where JROM increased until day 5 and then maintained a plateau or decreased thereafter.

A cross-correlation between joints is a measure of the similarity between the time series of two joint angles during a movement, with a value of 0 indicating that the increase/decrease in angles is occurring independently and a value of 1 indicating that they are changing perfectly in-phase. Thus, it has been proposed that a high cross-correlation is indicative of Bernstein’s second type of freezing – establishing temporary couplings between joints (McDonald et al., 1989). The predicted pattern of high cross-correlation between joints early in learning followed by a decrease with practice has been found in a study of dart throwing (McDonald et al., 1989) and the aforementioned studies of skiing (Vereijken et al., 1992) and soccer (Hodges et al., 2005).

There have also been two studies that have not found the predicted pattern of change in cross-correlation with learning. In a study using a balance task, Ko et al. (2003) found an increase in the cross-correlation with practice for the ankle–hip joints. Similarly, in their study of learning to pass a soccer ball, Chow et al. (2008) found that the cross-correlation values for the hip–knee and ankle–hip were lower pre-training than post-training. Guimarães et al. (2020) proposed that this discrepancy may be related to the goal of the task. Specifically, for tasks in which accuracy is emphasized, we might expect the reverse of Bernstein’s hypothesized pattern because freezing may be a means of increasing the precision of movement.

There are two additional recent studies that were not included in the review by Guimarães et al. (2020) because they used different measures for evaluating freezing. In a study by Ghorbani and Bund (2017) investigated the coordination of degrees of freedom involved in learning to a pitch a baseball. While previous studies have treated a movement skill as one single event, in this study, the skill was split into phases (windup, stride, arm cocking, arm acceleration, arm deceleration, and follow-through) and the coordination of degrees of freedom was evaluated separately for each phase. The authors predicted that more difficult phases of a movement (for example, ones that have faster movements or involve more body parts) would show more evidence of freezing of degrees of freedom that occurred earlier in acquisition. To test this, the kinematics of eight novices were compared to one expert baseball pitcher. Specifically, the normalized difference between the joint angles of the expert and novices were calculated for each phase. This difference was significantly higher for the stride phase of the delivery, which is the only one in which the pitcher is required to move the throwing arm and the striding leg at the same time. Qualitative analysis of the angle–angle plots for the elbow and shoulder suggested that this difference was due to a greater cross-correlation between joints for novices in the stride phase.

From the studies discussed so far, it can be seen that, depending on the task, freezing can take some time before it is implemented and may only be adopted for particular phases of a movement. Thus, it is reasonable to ask: is freezing a conscious control strategy on the part of the learner? The role of conscious processing in freezing was recently investigated by van Ginneken et al. (2018) using a seated ball throwing task. Prior to training, all participants were asked to complete the Movement Specific Reinvestment Scale (MSRS), which measures the predisposition to consciously control one’s movements (Masters et al., 2005). Participants were randomly split into an error-reduced (implicit) learning group that started throwing to a large target box that was gradually decreased in size and an error-strewn (explicit) learning group that always threw to the smallest target box. Following training, all participants completed two transfer tests: one involving a secondary task of counting tones (which reduces the ability to conscious control movements) and one involving performance pressure (which has been shown to increase conscious control). Consistent with idea that freezing is a conscious strategy, it was predicted that there would be greater evidence of freezing (as assessed by the variability in the movement of the throwing arm) for the error-strewn group and in the pressure transfer task and that these effects would be mediated by the predisposition to consciously control.

The data mostly supported the proposed link between conscious control and freezing. Overall, movement variability was significantly lower for the error-strewn group than for the error-reduced group. In the error-strewn group, propensity for conscious control was positively associated with both freezing (low variability) and throwing performance. In the error-reduced group, propensity for conscious control was negatively associated with performance, but not with freezing. Movement variability was significantly lower for the pressure transfer task than for the dual task transfer task. One limitation of this study is the coarse measure of freezing used (overall movement variability), which does not allow for evaluation of how the coordination of the degrees of freedom was achieved for the task.

The results from the study by van Ginneken et al. (2018) suggest that how the problem of movement coordination is solved during acquisition of a new skill may also depend on the instructional method (e.g., implicit vs. explicit learning). This effect was also found in a study investigating learning a forehand stroke in tennis (Lee et al., 2014). In this study, a linear instructional method (the use prescriptive repetitive drills) was compared to a non-linear method (manipulation of task constraints including the equipment and the rules). It was found that, even though there were no differences in performance, the non-linear training group showed greater movement variability and a greater number of movement patterns. The authors suggest that this may indicate that non-linear methods lead to a performer taking advantage of more of the available degrees of freedom in movement. Freezing and coupling were not assessed in this study, however.

To summarize, previous research has provided evidence largely consistent with Bernstein’s model of the coordination of degrees of freedom in acquiring a new skill. Measurements of JROM, movement variability, and the cross-correlation between joint angles have mostly provided support for the idea that performers initially freeze degrees of freedom and, with further practice, freeing occurs. However, how this is achieved and when it occurs seem to also depend on the complexity of the task, the phase of movement being considered, and the instructional method used.

In 2001, Newell and Vaillancourt identified some additional limitations with research in this area that are still applicable today. First, while Bernstein’s original hypotheses and the bulk of the studies in this area have focused on the biomechanical degrees of freedom of the joints, the issue of finding a coordination solution pertains to all levels of analysis of a movement system. Second, the evidence supporting Bernstein’s freezing/freeing hypothesis has been primarily descriptive in nature in that it has not been directly demonstrated that the freeing of degrees of freedom associated with accumulated practice trials necessarily represents a change in the coordination solution. Although, in the studies described above, it was found that performance outcome measures improved with practice along with the increases in JROM and reductions in cross-correlation between joints, no evidence has been provided that this freeing of degrees of freedom led to functional couplings or the development of coordinative structures. Finally, it is still an open question as to whether the progression from freezing to freeing degrees of freedom is a universal learning strategy or rather a consequence of the specific individual and task constraints involved. As described above, there is some evidence to suggest that how the degrees of freedom problem is solved is dependent on the specific task goals and constraints (e.g., the instructional method).

### Aims of the Present Study

The goal of the present study was to investigate movement coordination in baseball batting. In particular, the aims of the study were to (i) analyze the development of coordination at a different level of the system than has been examined in previous studies – in particular, the timing of the different phases of a complex movement; (ii) further investigate the relationship between the task constraints in the practice environment and the coordination of degrees of freedom; (iii) directly investigate the relationship between the coordination solution and performance (e.g., is freeing degrees of freedom with practice associated with the development of functional couplings?); and (iv) examine how a performer’s individual constraints influence the development of coordination.

To achieve this end, previously unanalyzed vertical ground reaction force (GRF_v_) data from a published training study (Gray, 2017) were used. Briefly, in this study, 80 participants (high school baseball players) were randomly assigned equally to groups undertaking adaptive hitting training in a virtual environment (VE), extra sessions of batting practice in the VE, extra sessions of real batting practice, and a control condition involving no additional training to the players’ regular practice. The adaptive training involved performance-based adjustments of pitch speed, pitch type, and location using staircase methods, and overall a larger variability in practice conditions. Training consisted of two 45-min sessions per week for 6 weeks. Performance on a batting test in the VE, in an on-field test of batting, and on a pitch recognition test was measured pre- and post-training. League batting statistics in the season following training and the highest level of competition reached in the following 5 years were also analyzed. For the majority of performance measures, the adaptive VE training group showed a significantly greater improvement from pre–post training as compared to the other groups. In addition, players in this group had superior batting statistics in league play and reached higher levels of competition.

**FIGURE 1:**
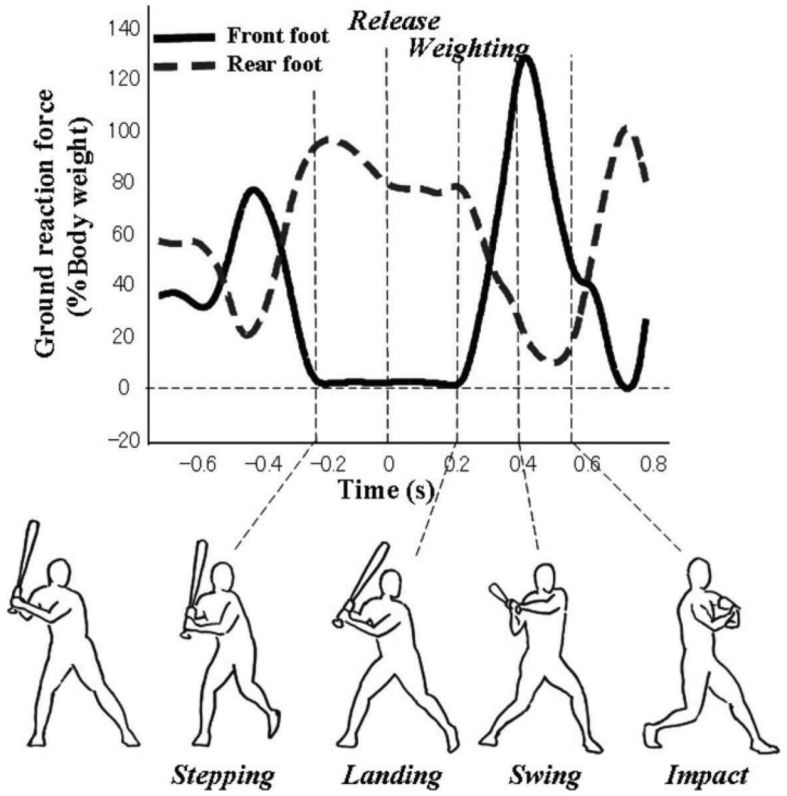
Ground reaction forces for different phases of a baseball swing. Reproduced with permission from Katsumata (2007).

To understand how the movements involved in the swing were coordinated for the different training groups in the Gray (2017) study, vertical GRF_v_ were analyzed using the approach developed by Katsumata (2007). In his study, a baseball swing was divided into five distinct phases using GRF and motion tracking as shown in Figure 1:

(1) *Stepping*: Time at which the front foot (i.e., closest to the oncoming ball) GRF_v_ is zero, corresponding to front foot leaving the ground.
(2) *Landing*: Time at which the front foot GRF_v_ became larger than zero, indicating it has returned to the ground.
(3) *Weighting:* Time at which the rate of change of front foot GRF_v_ surpassed 50% of the hitter’s body weight, indicating a shift in weight to the front foot.
(4) *Swing:* Time at which the bat started moving forward, as identified from motion tracking.
(5) *Impact:* Time at which the bat made contact with the ball.

Coordinating the different phases of this complex movement in a manner that leads to a swing that is both powerful and timed appropriately for the trajectory of the ball represents a form of the degrees of freedom problem for the batter (Katsumata, 2007). Specifically, there are multiple possible combinations of timing and duration that could be used, e.g., some phases could be held constant from swing–swing, others could be functionally coupled, etc. How is the problem of coordination solved in this situation?

In Katsumata’s study, college baseball batters hit balls launched by a pitching machine in fast (32 m/s; 72 m/h) and slow (20 m/s; 45 m/h) pitch conditions. Analysis of the GRF_v_ data revealed that the timing of the forward weight shift relative to the release of the pitch (*Weighting*) was the primary aspect of the swing that was modulated to adjust for different pitch speeds. In particular, for the slow pitches, batters remained momentarily with their weight on their rear foot, delaying a bit the start of the weight shift to the front foot. Furthermore, there was strong evidence for functional coupling between the different phases of the movement. In particular, there was a significant negative correlation between the timing of *Weighting* and the *Weighting-Swing* duration. So, in other words, if the batter started their forward weight shift a bit later (relative to pitch release), this was compensated for by having a shorter duration between this weight shift and the start of the swing. Similar results were found for the relationship between *Landing* and the *Landing-Swing* duration. Finally, further evidence for this functional coupling between the different phases of movement (i.e., a coordinative structure) came from an analysis of the variability of each of the swing phases shown in Figure 1. It was found that there was a significant decrease in variability between phases occurring earlier (e.g., Stepping) and later in the movement (e.g., Impact), which could only occur if the swing was being functionally adjusted online as it unfolds.

Similar findings have been reported for other measures of batting performance. Nakata et al. (2013) compared surface electromyography (sEMG) in the lower extremities for skilled (college) baseball players and unskilled novices hitting soft toss thrown by coach. The timing of the different swing movements was measured using high-speed video. There was a significant difference in how the timing of the different phases of the swing was coordinated for the two groups. The onset of the sEMG and the stepping and landing phases occurred earlier for the skilled group while the onset of the swing phase occurred earlier for the unskilled group. In addition, there was a double peak in the sEMG measured at the tibialis anterior of the front leg of all 10 skilled batters but only five of the unskilled batters, indicating a different coordination of the weight shit and timing of the swing.

Nakata et al. (2012) compared head movements (measured using high speed video) for eight skilled (college) baseball players and nine unskilled novice, again hitting soft toss. The timing of the change in direction of the head movement from backwards (away from the ball) to forwards occurred significantly earlier and was less variable for the skilled batters. Furthermore, for 6/8 of the skilled batters, the forwards movement of the head stopped temporally at the instant of bat–ball impact (indicating they were waiting for the ball), while this was observed for only one of the unskilled batters. These findings again suggest that coordination of timing of the different movements involved in batting are one the main things that changes as a batter becomes more experienced.

In the present study, we first performed a similar set of analyses to Katsumata (2007) using the GRF data from Gray (2017). Of particular interest was how the variability of timing of the different swing stages and the strength of the functional couplings described above might change from pre and post training for the different training groups. However, to extend on this and look more specifically at how it relates to batting performance, an analysis conceptually based on the uncontrolled manifold (UCM) hypothesis (reviewed in Latash, 2012) was also used, as described next.

Since the timing of the *Weighting* event was one of the main parts of the swing that was adjusted for different pitches in Katsumata (2007), in the present study, the swing was divided into two phases centered around this event:

(1) *Release-Weighting:* The time between the ball leaving the virtual pitcher’s hand and the shift of the weight to the front foot.
(2) *Weighting-Bat in Hitting Zone:* The time between the shift of the weight to the front foot and the instant in time where the bat reached the contact area. Note, as discussed below, this variable was used instead of *Impact* so that swings for which the batter missed the ball could also be included.

**FIGURE 2:**
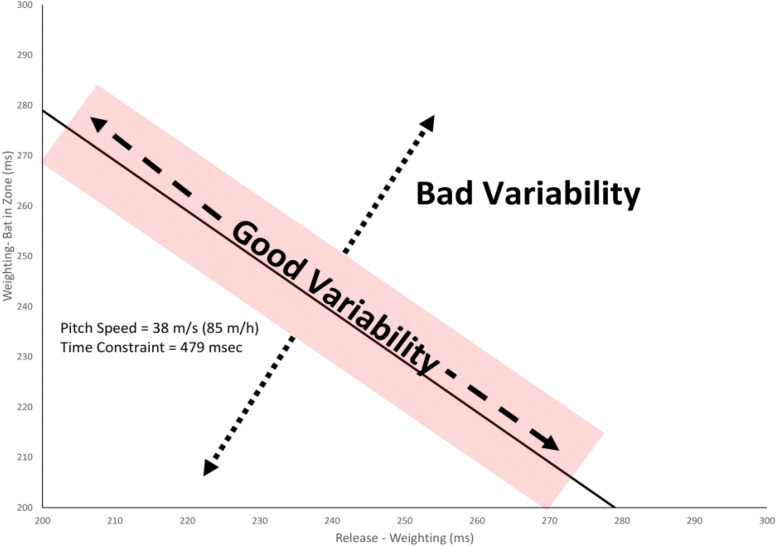
Partioning variability in swing timing into good variability (any change that keeps the total swing time within the temporal constraint) and bad variability (any change in timing that makes the swing time less or more than the temporal constraint). The shaded area shows the estimated margin for error for getting a hit of ± 10 ms (Watts and Bahill, 2000).

The swing phase was split into these two phases in the present study to allow for an analysis of the relationship between the coordination of movement and the performance outcome. As illustrated in Figure 2, baseball batting imposes a temporal constraint on the hitter that the *Release-Weighting* plus *Weighting-Bat in Hitting Zone* durations must sum to equal the flight time of the ball (plus or minus some margin for error) for a successful hit. Following the UCM hypothesis, it is then possible to separate “good” vs. “bad” variability in the swing. Good variability is any change in the swing coordination which keeps the temporal constraint satisfied. This type of variation is considered to be good because it allows the swing to be adaptable to variations in the internal state of the batter and the external environment. Bad variability is any change in the coordination between these two parts of the swing that causes the temporal constraint not to be satisfied (i.e., the bat gets to the hitting area too late). In the present study, the amount of good and bad variability was compared pre–post training and for different pitch types in the Gray (2017) study to evaluate to what extent any changes in coordination were functional in terms of performance outcomes.

Finally, as discussed above, it has been proposed that the manner in which the degrees of freedom problem is solved is dependent on not only the constraints of the task being performed but also the individual constraints of the performer (Newell and Vaillancourt, 2001). To test this idea, the maximum bat speed for each batter was measured pre-training. In a previous study, it was found that this variable was significantly related to the manner in which batter’s adapted to a change in the task constraints – an increase in bat weight (Scott and Gray, 2010). Specifically, hitters that could generate a higher bat speed were more able to adjust their movement pattern to increases in bat weight. In the present study, the relationship between this individual constraint and the timing of the different swing phases was assessed.

### Hypotheses Tested in the Present Study

There were two overall predictions tested in the present study. First, consistent with Bernstein’s concept of freeing degrees of freedom, it was hypothesized that all the training groups from the Gray (2017) study would show more evidence of functional coupling between the different swing phases and good variability in the swing in post-training tests as compared to pre-training. Second, it was hypothesized that the magnitude of these changes would be significantly greater for batters in the VE Adaptive group as compared to the other training groups. This latter prediction was based both on the *a priori* knowledge that this group performed better on tests of hitting and reached higher levels of competition in the Gray (2017) study and on the finding discussed above that non-linear instruction methods with higher variability result in the learner taking advantage of more of the available degrees of freedom in movement (Lee et al., 2014). This resulted in the following of set of specific hypotheses.

(i) The decrease in the variability of the swing phases from early in the swing (*Landing*) to late in the swing (*Bat in Hitting Zone*) would be significantly greater post-training as compared to pre-training.
(ii) The correlation between *Landing* and *Landing-Swing* would be significantly more negative post training.
(iii) The correlation between *Weighting* and *Weighting-Swing* would be significantly more negative post training.
(iv) The amount of good variability in the swing would significantly increase from pre–post training.
(v) The amount of bad variability in the swing would significantly decrease from pre–post training.
(vi) All of the effects described above would be significantly greater for the VE Adaptive group as compared to the other training groups.
(vii) Across all training groups, the magnitude of the effects described above would be correlated with the measures of hitting performance (e.g., # of hits) used in Gray (2017).
(viii) Batters with a higher bat speed be would have more flexibility in their coordination solution their movement. Therefore, across all training groups, the magnitude of the effects described above would be correlated with the maximum bat speed.

## Materials and Methods

### Apparatus and Procedure

The GRF data used for the present study were collected in the VE batting tests in Gray (2017). The batting VE has been using in several previous studies (e.g., Gray, 2002, 2004, 2009) and was composed of a large screen projection of a simulated approaching ball, pitcher, and playing field. Batters attempted to hit the virtual ball with a real bat (Rawlings “Big Stick” Professional Model, 84 cm) equipped with a motion tracking sensor (Polhemus Fastrack, 120 Hz). See Gray (2017) for more details.

GRFs were measured using two OPTIMA Biomechanics Measurement Series (BMS) 0.6 (W) × 0.6 (L) × 0.1 (H) m force plates (Advanced Medical Technology Inc., Watertown, MA, United States). The plates were placed directly beside each other in the batters’ box. Batters were asked to stand with their back foot (furthest from the pitcher) near the edge of the back plate and place their lead foot in whatever position was comfortable for them. Note that the dimensions of the plates were roughly equivalent to the required size based on calculations of stance and stepping length during hitting (Katsumata, 2007) and, indeed, during data collection, it was observed that none of the batters came off the force plates while swinging.

In the VE hitting tests, batters faced a series of pitches until the sum of the number of strikes plus the number of hits was equal to twenty. The lateral location and height of each pitch were varied such that 65% of the pitches crossed the plate in the strike zone. Pitch type (fastball, curveball, or changeup) was chosen randomly on each pitch. These specifics of these pitch types were: (i) a “four seam” fastball with a speed of 85 mph (38 m/s), thrown with backspin, and with a spin rate of 1900 rpm, (ii) a “12–6” curveball with a speed of 65 mph (29.0 m/s), thrown with topspin, and with a spin rate of 1700 rpm, and (iii) a “straight change” with a speed of 70 mph (31.2 m/s), thrown with backspin, and with a spin rate of 1800 rpm. Only trials for which the batter swung the bat (defined as the bat crossing the front edge of the plate) were used in the analyses described below. See Gray (2017) for further details.

The maximum bat speed was measured prior to the beginning of the pre-tests. Batters were asked to try to “hit the virtual ball as hard as possible” for a fastball thrown to the middle of the strike zone. This was repeated five times and the average of the bat speeds at the instant the bat reached the hitting zone (described below) was calculated.

### Data Analysis

Following Katsumata (2007), the force plate data were filtered with a cutoff frequency of 10 Hz using a second-order Butterworth filter and standardized by dividing the force value by the body weight of a corresponding participant. The phases of the swing were calculated using the definitions described above (Katsumata, 2007). The time at which the bat reached the hitting zone was defined as the point at which the attack angle became positive, i.e., when the bat start moving upwards after reaching its minimum height in swing (Gray, 2018).

As illustrated in Figure 2, good variability was defined as any changes in the timing of the *Weighting* and *Weighting-Bat in Hitting Zone* swing phases that kept the total swing time equal to the temporal constraint plus/minus the margin for error. Bad variability was defined as changes in the timing of these swing phases that resulted in a swing time that was outside of this range. To calculate these values, the distance of each swing from a reference point was first calculated. For good variability, this involved taking all data points (*x*,*y*) that fell within the temporal constraint (plus/minus the margin for error) for the given pitch speed and calculating the distance from the mean value of *Weighting* and *Weighting-Bat in Hitting Zone times* for all such swings using the formula:

(1) $$d_{g}=\sqrt{{\left(x-\bar{x}\right)}^{2}+{\left(y-\bar{y}\right)}^{2}}$$

For bad variability, the perpendicular distance (*d*) of each data point (*x*, *y*) from the temporal constraint line was calculated for all swings that fell outside the temporal constraint region using the formula:

(2) $$d_{b}=\frac{\left|x+y-C\right|}{\sqrt{2}}$$

where *C* is the time between the point of release and the ball reaching the hitting zone (i.e., the temporal constraint), *x* is the *Release-Weighting* time, and *y* is the *Weighting-Bat in Zone* time. So, for example, for the 85 m/h pitch illustrated in Figure 2, this formula calculates the perpendicular distance of a given data point from the line *y* = 479 − *x*. Finally, the standard deviation of *d*_g_ and *d*_b_ were calculated and used as the estimates of good and bad variability, respectively.

Prior to performing the ANOVA and correlation analyses, all data were checked, including statistical outliers and normality, using the Kolmogorov–Smirnov test. If normality was confirmed, Pearson’s correlation was calculated; otherwise, Spearman’s correction was used.

For some of the dependent variables, data were analyzed separately for fastball and changeup pitch types. Due to the randomization procedure used in Gray (2017), not all batters faced the same types of pitches. However, there were no significant differences (*p* > 0.05) between the number of each pitch type faced in the pre- and post-tests. Mean and standard deviation for the number of each pitch type were: FB/Pre-test, 6.1 (0.77), FB/Post-test, 6.3 (0.88), CU/Pre-test, 6.2 (0.76), and CU/Post-test, 5.9 (0.74).

## Results

### Variability in the Timing of the Swing Phases

**FIGURE 3:**
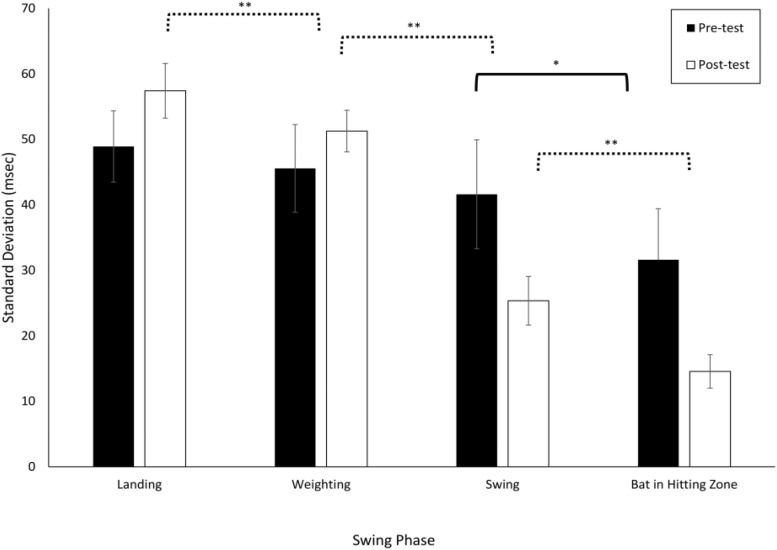
Standard deviations of the timing of the different swing phases defined by Katsumata (2007). Data are means for all batters in the VE Adaptive training group from Gray (2017). ^∗^*p* < 0.05, ^∗∗^*p* < 0.01.

Figure 3 shows the mean and standard deviation (averaged across all participants) for the VE Adaptive group in the pre- and post-training batting tests. From this figure, it can be seen that there was more online adjustment of the swing (i.e., a greater reduction in variability with each subsequent phase) post-training as compared to pre-training. These data were first analyzed using a 2 × 4 × 4 Mixed ANOVA with Test Period (Pre, Post) and Swing Phase (Landing, Weighting, Swing, Bat in Zone) as within-subject factors and Training Group as the between-subject factor. This analysis revealed that all main effects and interactions were significant: group, *F*(3, 76) = 7.0, *p* < 0.001, $\eta_{\text{p}}^{2}$ = 0.22; test period, *F*(1, 76) = 1244.8, *p* < 0.001, $\eta_{\text{p}}^{2}$ = 0.94; phase, *F*(3, 228) = 625.3, *p* < 0.001, $\eta_{\text{p}}^{2}$ = 0.89; group × test period, *F*(3, 76) = 13.4, *p* < 0.001, $\eta_{\text{p}}^{2}$ = 0.35; group × phase, *F*(9, 228) = 4.1, *p* < 0.001, $\eta_{\text{p}}^{2}$ = 0.14; test period × phase, *F*(3, 228) = 21.5, *p* < 0.001, $\eta_{\text{p}}^{2}$ = 0.22; and group × test period × phase, *F*(9, 228) = 2.8, *p* = 0.003, $\eta_{\text{p}}^{2}$ = 0.10. Following Katsumata (2007), pairwise *t*-tests (with Bonferroni correction, *p* = 0.008) were next used to compare adjacent swing phases. For the VE Adaptive group in the pre-test, the only significant difference was between the final two phases, *Swing* and *Bat in Hitting Zone* [*t*(19) = 3.8, *p* < 0.001, *d* = 1.2]. In the post-test, the standard deviation for *Weighting* was significantly lower than that for *Landing* [*t*(19) = 7.6, *p* < 0.001, *d* = 1.6], *Swing* was significantly lower than *Weighting* [*t*(19) = 15.1, *p* < 0.001, *d* = 5.4], and *Bat in Hitting Zone* was significantly lower than *Swing* [*t*(19) = 6.1, *p* < 0.001, *d* = 3.4].

Table 1 shows comparable values for the other training groups in the Gray (2017) study. The variability in adjacent swing phases was compared separately for each group using pairwise *t*-tests. In the pre-test, all four groups had a significant decrease in standard deviation in the final two phases, *Swing* to *Bat in Hitting Zone*. For the Real BP and Control groups, there was also a significant decrease from the *Weighting* to *Swing* phase. In the post-test, the pattern of results was similar for all the training groups, except that only the VE Adaptive group had a significant difference in standard deviation between the *Landing* and *Weighting* phases. Thus, the results provide partial support for hypothesis (i) – the reduction in variability from the start to end of the swing seemed to increase with training for the two VE training groups but not for the Real BP or Control groups. The results also provide some support for hypothesis (vi) as only the VE Adaptive group showed a significant decrease in variability between the *Landing* and *Weighting* phases post-training.

**TABLE 1:** Mean and standard deviations of the different swing phases.

Group/Test period	Landing	Weighting	Swing	Bat in zone
VE_Adaptive/Pre	50.9 (5.7)	47.5 (6.9)	45.7 (8.4)	31.6 (7.8)*
VE_BP/Pre	50.8 (5.9)	48.6 (6.0)	48.4 (4.7)	29.4 (5.0)*
Real_BP/Pre	53.8 (6.2)	53.0 (5.5)	47.3 (5.1)*	32.4 (6.3)*
Control/Pre	51.1 (7.1)	50.1 (6.3)	44.6 (5.2)*	33.0 (5.5)*
VE_Adaptive/Post	57.4 (4.2)	51.2 (3.2)*	25.3 (3.7)*	14.6 (2.5)*
VE_BP/Post	52.8 (7.6)	50.4 (6.9)	38.3 (4.8)*	19.1 (4.1)*
Real_BP/Post	55.7 (6.1)	54.1 (7.0)	37.7 (4.7)*	20.5 (5.0)*
Control/Post	47.8 (7.8)	46.6 (6.6)	31.3 (5.3)*	23.1 (4.4)*

*Significantly lower than preceding swing phase, p < 0.01. Values in parenthesis are SD. VE_Adaptive, adaptive training with higher variability; VE_BP, extra low variability batting practice in the VE; Real_BP, extra low variability batting practice in a batting cage; Control, no additional training.

From Figure 3, it can also be seen that there was some evidence of Bernstein’s hypothesized progression from freezing to freeing degrees of freedom in movement. Specifically, consistent with the idea of freeing and the movement variability results of van Ginneken et al. (2018) described above, the variability of the timing of the *Landing* [*t*(19) = 5.4, *p* < 0.001, *d* = 0.9] and *Weighting* [*t*(19) = 3.5, *p* = 0.002, *d* = 0.8] phases of the swing increased significantly from pre–post training for the VE Adaptive group. Although, as shown in Table 1, this pattern was also seen for the other training groups, the difference was not statistically significant.

### Coupling Between Swing Phases

**FIGURE 4:**
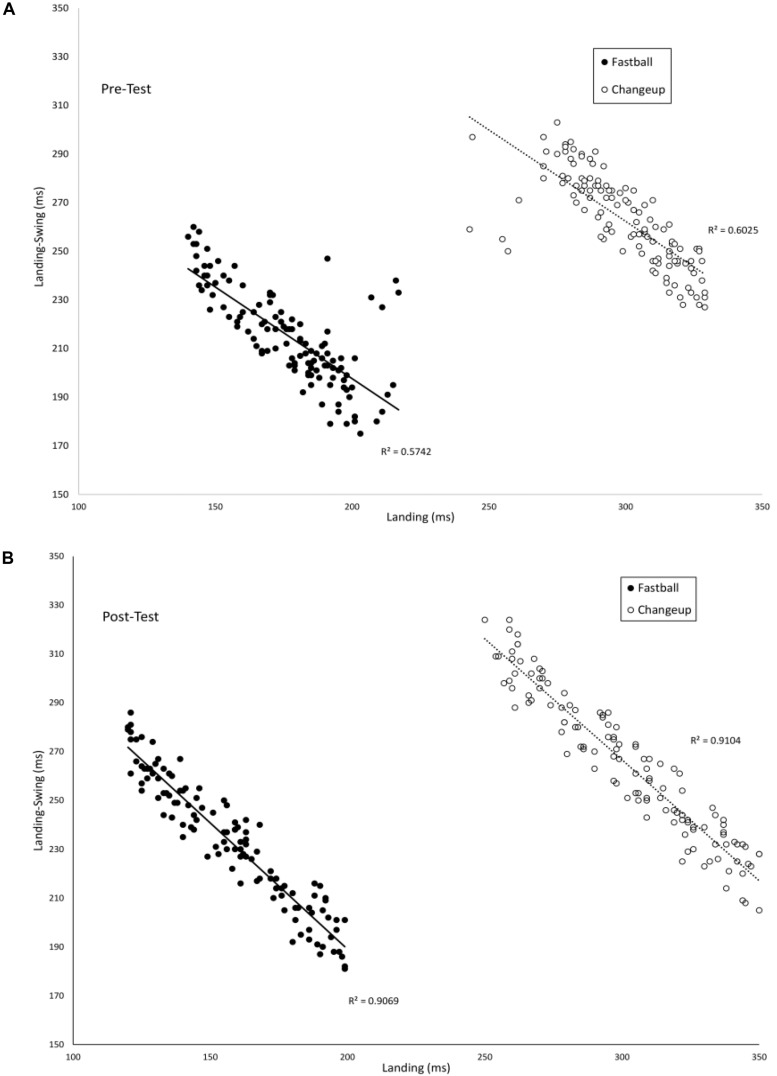
Timing of the *Landing* and *Landing-Swing* phases of the swing as measured from the instant of pitch release.

Figure 4 plots the timing of the *Landing* phase vs. the duration between *Landing-Swing* for batters in the VE Adaptive group in the pre-test (A) and post-test (B). Each data point in the figure shows one swing. To allow for direct comparison with Katsumata (2007), data were analyzed separately for fastballs and changeups. In the pre-test, there was a significant negative correlation between *Landing* and *Landing-Swing* for both fastball [*r*(118) = −0.76, *p* < 0.001] and changeup [*r*(118) = −0.78, *p* < 0.001] pitch types. In the post-test, these correlations were again significant: fastball [*r*(118) = −0.95, *p* < 0.001] and changeup [*r*(118) = −0.94, *p* < 0.001]. For both pitch types, the correlation in the post-test was significantly larger in magnitude as compared to the pre-test: fastball (*z* = 6.39, *p* < 0.001) and changeup (*z* = 5.3, *p* < 0.001). Table 2 shows comparable values for the other training groups in the study. Consistent with hypothesis (ii), for all groups except the control group, the correlation between the *Landing* and *Landing-Swing* phases was significantly greater in magnitude in the post-test as compared to the pre-test.

**TABLE 2:** Correlations between the *Landing* and *Landing-Swing* phases.

Group/Pitch type	Pre-test	Post-test
VE_Adaptive/FB	–0.76	−0.95*
VE_BP/FB	–0.81	−0.9*
Real_BP/FB	–0.74	−0.91*
Control/FB	–0.79	–0.86
VE_Adaptive/CU	–0.78	−0.95*
VE_BP/CU	–0.75	−0.88*
Real_BP/CU	–0.82	−0.92*
Control/CU	–0.77	–0.85

*Significantly different in the pre- and post-tests, p < 0.01. VE_Adaptive, adaptive training with higher variability; VE_BP, extra low variability batting practice in the VE; Real_BP, extra low variability batting practice in a batting cage; Control, no additional training.

**FIGURE 5:**
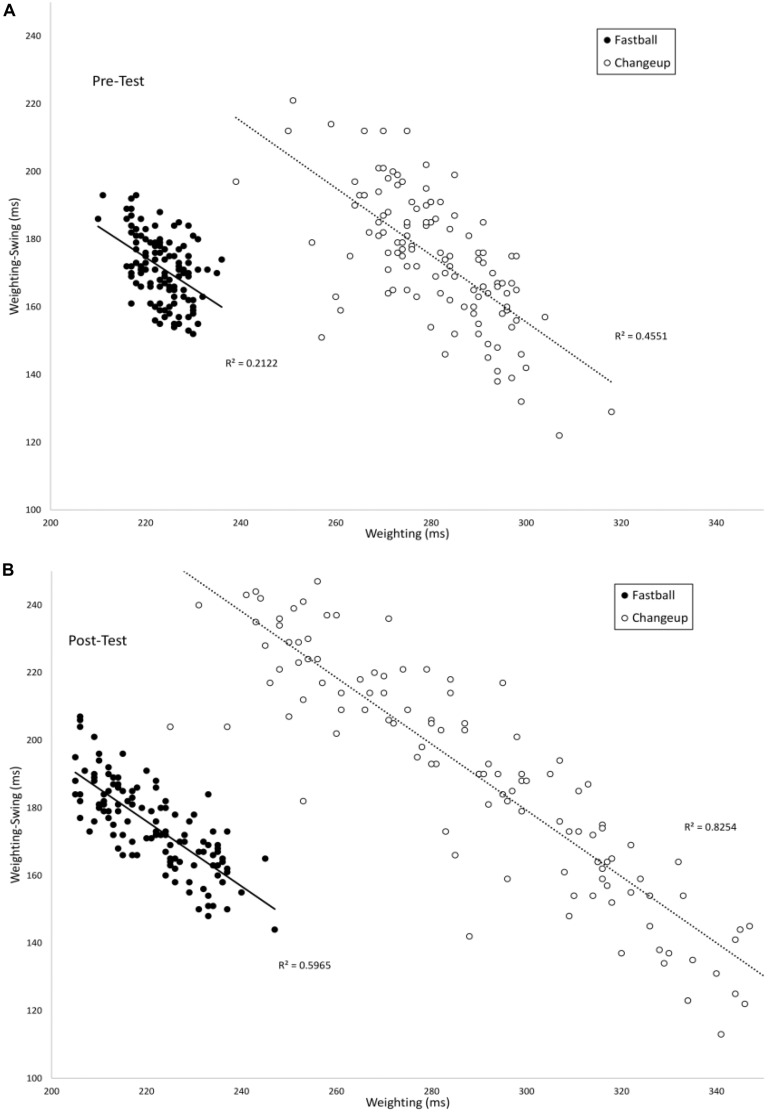
Timing of the *Weighting* and *Weighting-Swing* phases of the swing as measured from the instant of pitch release.

Figure 5 plots the timing of the *Weighting* phase vs. the duration between *Weighting-Swing* for batters in the VE Adaptive group in the pre-test (A) and post-test (B). In the pre-test, there was a significant negative correlation for both fastball [*r*(118) = −0.46, *p* < 0.001] and changeup [*r*(118) = −0.68, *p* < 0.001] pitch types. In the post-test, these correlations were again significant: fastball [*r*(118) = −0.77, *p* < 0.001] and changeup [*r*(118) = −0.90, *p* < 0.001]. For both pitch types, the correlation in the post-test was significantly larger in magnitude as compared to the pre-test: fastball (*z* = 4.0, *p* < 0.001) and changeup (*z* = 4.9, *p* < 0.001). Table 3 shows comparable values for the other training groups in the study. In this case, the difference between correlations in pre- and post-tests was only significant for the VE Adaptive group.

**TABLE 3:** Correlations between the *Weighting* and *Weighting-Swing* phases.

Group/Pitch type	Pre-test	Post-test
VE_Adaptive/FB	–0.46	−0.77*
VE_BP/FB	–0.20	−0.47*
Real_BP/FB	–0.18	−0.42*
Control/FB	–0.15	−0.50*
VE_Adaptive/CU	–0.68	−0.90*
VE_BP/CU	–0.41	–0.35
Real_BP/CU	–0.38	–0.36
Control/CU	–0.37	–0.28

*Significantly different in the pre- and post-tests, p < 0.05. VE_Adaptive, adaptive training with higher variability; VE_BP, extra low variability batting practice in the VE; Real_BP, extra low variability batting practice in a batting cage; Control, no additional training.

From Figures 4, 5, there again appears to be evidence of freeing degrees of freedom with training. Specifically, in the post-test, participants in the VE Adaptive group used a wider range of timings of the different swing phases as compared to the pre-test. To evaluate this, the mean ranges for *Landing* and *Weighting* in the pre- and post-test were compared using pairwise *t*-tests. Ranges were significantly larger for both phases in the post-test: *Landing*, Pre-test (*M* = 47.4, *SE* = 12.8), Post-test (*M* = 66.9, *SE* = 16.4), *t*(19) = 3.7, *p* = 0.002, *d* = 1.3; *Weighting*, Pre-test (*M* = 12.4, *SE* = 4.3), Post-test (*M* = 32.1, *SE* = 13.0), *t*(19) = 5.5, *p* < 0.001, *d* = 2.0.

### Good vs. Bad Variability

**FIGURE 6:**
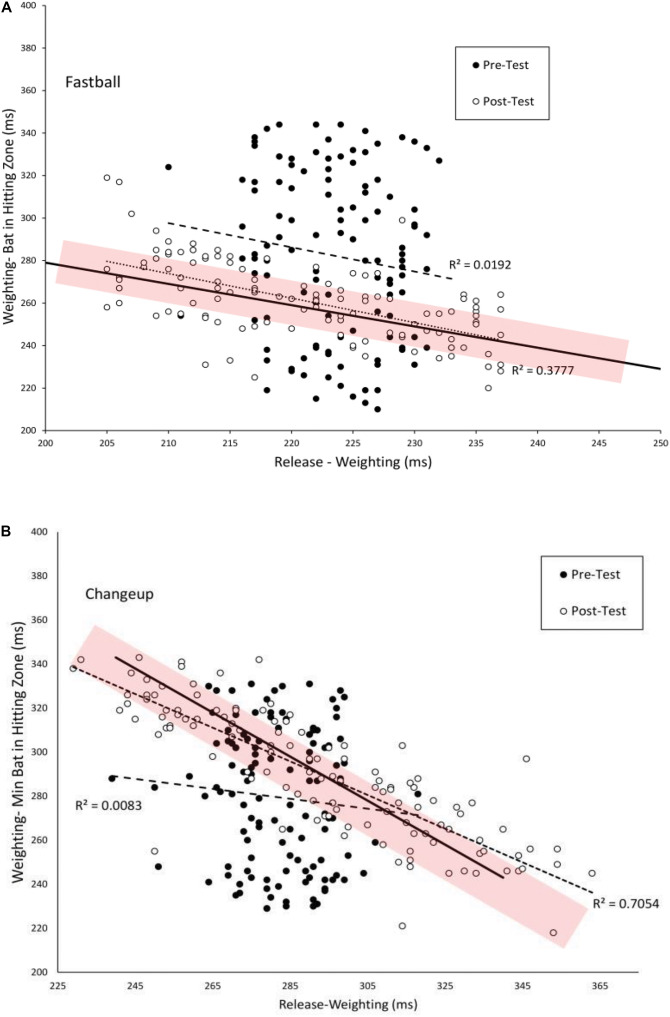
Timing of the *Weighting* and *Weighting-Bat in Hitting Zone* phases of the swing as measured from the instant of pitch release. The solid lines show combination of the two phases that are equal to the total flight time of the pitch. The shaded area shows the estimated margin for error for getting a hit of ± 10 ms (Watts and Bahill, 2000). Dashed and dotted lines are linear curve fits to the data.

Figure 6 plots the duration between *Weighting-Bat in Zone* as a function of *Weighting* for batters in the VE Adaptive group for fastballs (A) and changeups (B). In the pre-test, these swing phases were not significant correlated for fastballs (*r* = −0.14) or changeups (*r* = −0.09). In the post-test, there was a significant negative correlation between these swing phases for both fastballs [*r*(118) = −0.61, *p* < 0.001] and changeups [*r*(118) = −0.84, *p* < 0.001]. Thus, it appears that a functional coupling between these two phases of the swing was developed with training. Table 4 shows comparable values for the other training groups in the study. For all four groups, the magnitude of the correlation was significantly higher in the post-test for fastballs while, for changeups, the pre–post difference was only significant for the VE Adaptive group.

**TABLE 4:** Correlations between the *Release-Weighting* and *Weighting-Bat in zone* phases.

Group/Pitch type	Pre-test	Post-test
VE_Adaptive/FB	–0.46	−0.78*
VE_BP/FB	–0.52	–0.66
Real_BP/FB	–0.49	–0.64
Control/FB	–0.55	–0.70
VE_Adaptive/CU	–0.57	−0.90*
VE_BP/CU	–0.53	–0.69
Real_BP/CU	–0.62	–0.75
Control/CU	–0.54	–0.65

*Significantly different in the pre- and post-tests, p < 0.01. VE_Adaptive, adaptive training with higher variability; VE_BP, extra low variability batting practice in the VE; Real_BP, extra low variability batting practice in a batting cage; Control, no additional training.

As can also be seen in Figure 6, from pre–post test, there was an increase in the number and variability of swings that fell within the shaded temporal constraint area and a decrease in the number and variability of swings that resulted in the total swing time being above or below the constraint. Table 5 shows the mean good and bad variability estimates for the four training groups. For the VE Adaptive group, there was a significant increase in good variability [*t*(19) = 8.2, *p* < 0.001, *d* = 2.8] and a significant decrease in bad variability [*t*(19) = −13.3, *p* < 0.001, *d* = 4.2] from pre–post training for fastballs. Similar results were also found for changeups: good variability [*t*(19) = 2.5, *p* = 0.02, *d* = 0.7] and bad variability [*t*(19) = 7.8, *p* < 0.001, *d* = 2.2]. For the three other groups in the study, there was also a significant decrease in bad variability from pre–post training for fastballs. The changes in good variability and change in bad variability for changeups were not significant for the other training groups.

**TABLE 5:** Good and bad variability estimates.

Group/Pitch type	Good pre-test	Post-test	Bad pre-test	Post-test
VE_Adaptive/FB	5.7	12.9*	27.9	8.4*
VE_BP/FB	9.9	10.2	20.2	14.3*
Real_BP/FB	7.7	8.2	29.8	17.6*
Control/FB	8.2	9.0	23.3	11.2*
VE_Adaptive/CU	32.9	38.7*	22.5	9.6*
VE_BP/CU	25.6	24.4	18.7	16.5
Real_BP/CU	35.5	38.8	26.5	25.1
Control/CU	30.8	33.6	19.7	17.7

*Significantly different in the pre- and post-tests, p < 0.01. VE_Adaptive, adaptive training with higher variability; VE_BP, extra low variability batting practice in the VE; Real_BP, extra low variability batting practice in a batting cage; Control, no additional training.

### Relationship Between Swing Timing and Hitting Performance

**FIGURE 7:**
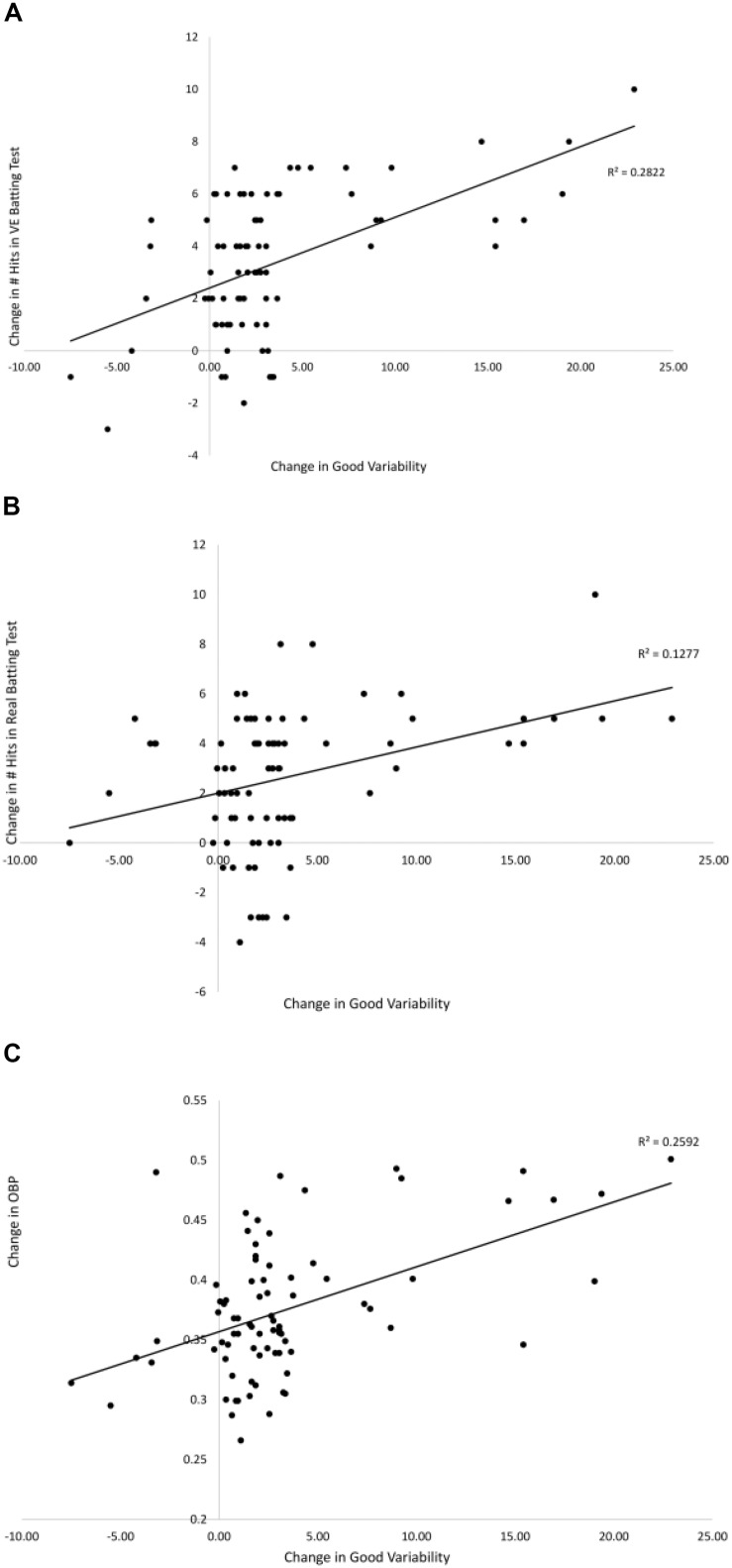
Relationships between the pre–post change in good variability and the pre–post changes in: # hits in the VE hitting test **(A)**, # hits in the Real hitting test **(B)**, and OBP from season play **(C)** from Gray (2017). Solid lines are linear curve fits.

**FIGURE 8:**
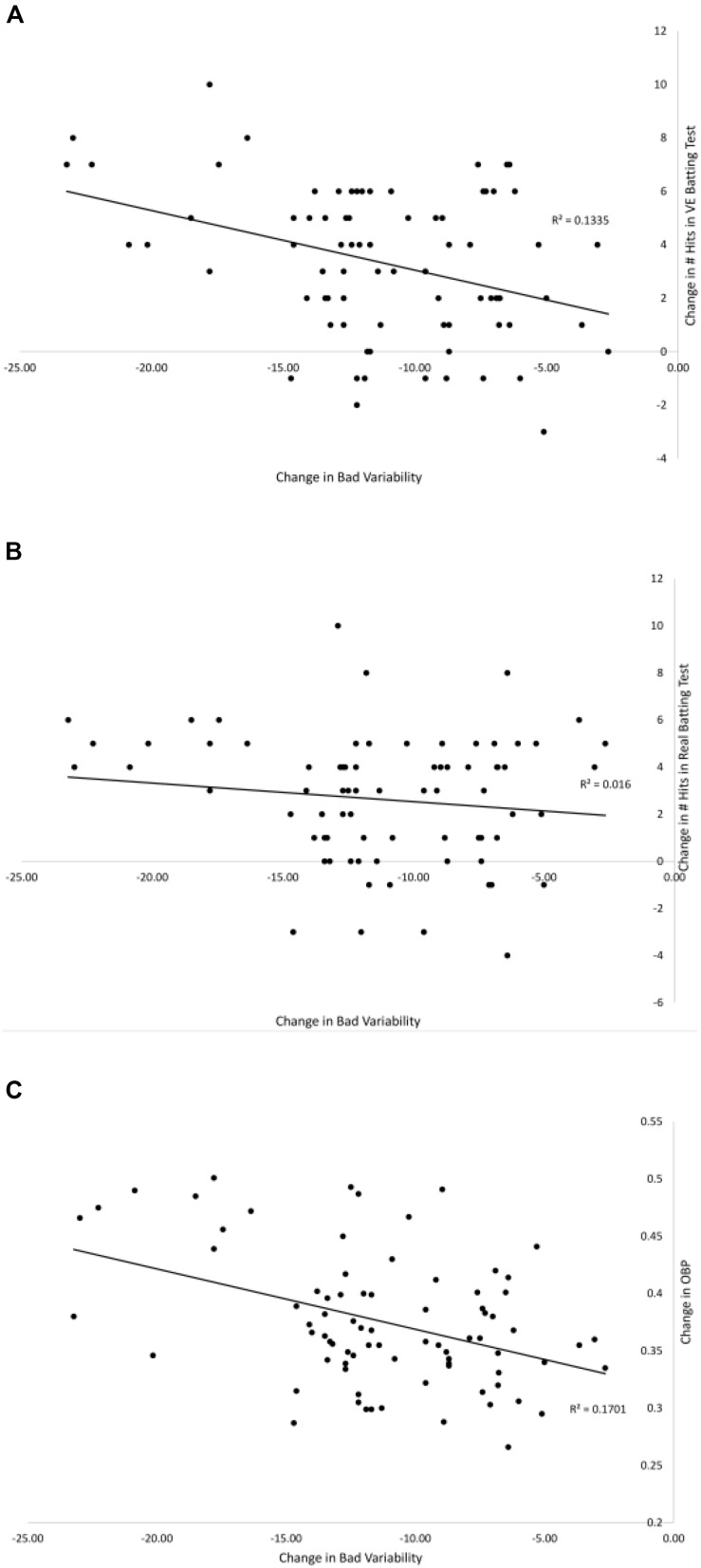
Relationships between the pre–post change in bad variability and the pre–post changes in: # hits in the VE hitting test **(A)**, # hits in the Real hitting test **(B)**, and OBP from season play **(C)** from Gray (2017). Solid lines are linear curve fits.

Figures 7, 8 show the relationships between pre–post changes in good and bad variability and the pre–post changes in three performance outcome measures for all participants in the Gray (2017) study. On-base percentage (OBP) is a measure of how often a player reaches base with the exact formula, OBP = (Hits + Walks + Hit by Pitch)/(At Bats + Walks + Hit by Pitch + Sacrifice Flies). As can be seen in the figures, batters that had a larger increase in good variability after training had significantly more hits in the VE batting test [*r*(78) = 0.53, *p* < 0.01], more hits in Real batting test [*r*(78) = 0.35, *p* = 0.01], and significantly higher OBP in the season following the training [*r*(78) = 0.49, *p* < 0.01]. There were also significant negative correlations found between the change in the amount of bad variability and the number of hits in the VE [*r*(78) = −0.37, *p* = 0.01] and OBP [*r*(78) = −0.41, *p* < 0.01]. The correlation between change in bad variability and change in number of Real hits (-0.13) was not significant.

### Relationship With Maximum Bat Speed

**FIGURE 9:**
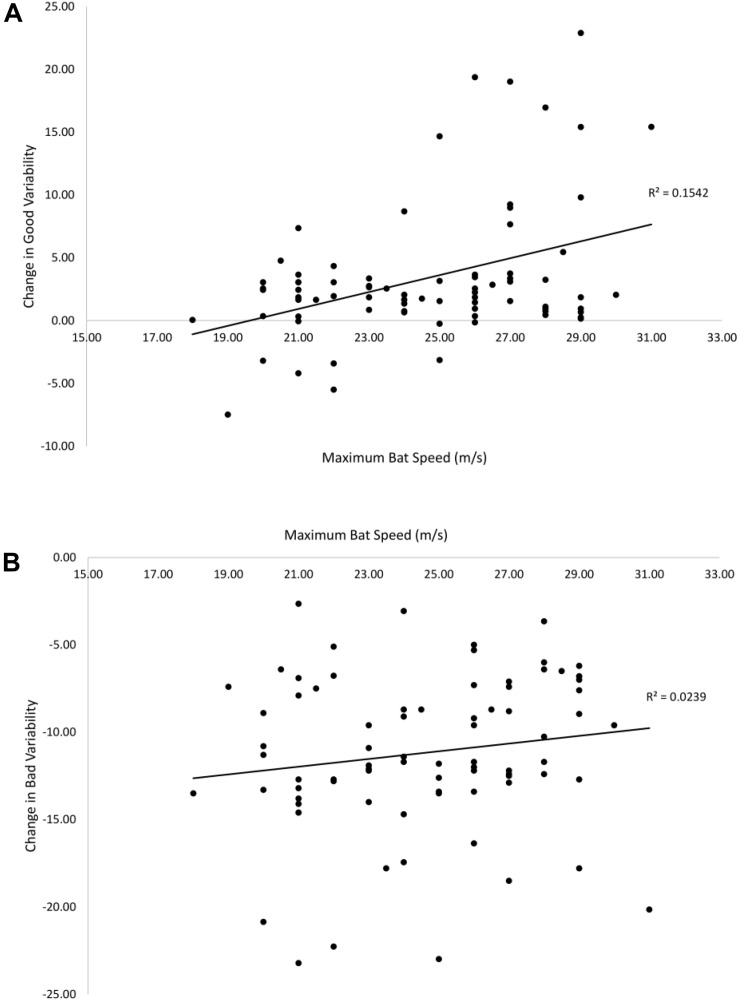
Relationships between maximum bat speed and the pre–post change in good **(A)** and bad **(B)** variability. Solid lines are linear curve fits.

Figure 9 shows the relationship between the estimated maximum bat speed and pre–post change in good (A) and bad (B) variability. There was a significant positive correlation between bat speed and change in good variability [*r*(78) = 0.39, *p* < 0.01]. The correlation between bat speed and change in bad variability (-0.15) was not significant. Therefore, being able to generate a higher bat speed was associated with a greater change in good variability following training.

## Discussion

Coordinating the timing of the different phases of a baseball swing in a manner that leads to a swing that is both powerful and timed appropriately for the trajectory of the oncoming ball represents a form of Bernstein’s degrees of freedom problem for the batter. Previous research has shown that this coordination problem is solved in skilled (college) batters through the use of coordinative structures or functional couplings between the different swing phases (Katsumata, 2007). The goal of the present study was to investigate how these coordinative structures develop through training and how this development depends on the structure of practice and the batter’s individual constraints. I first examine results for the group that showed the greatest performance changes in the Gray (2017) study (VE Adaptive) followed by a discussion of differences between the training groups.

A first question to consider is to what extent batters in the present study exhibited the hypothesized progression from freezing to freeing degrees of freedom proposed by Bernstein (1967). Consistent with a systematic review discussed above (Guimarães et al., 2020), the overall pattern of results in the present study provides some support for this hypothesis. For batters in the VE Adaptive group from the Gray (2017) study, the improvement in batting performance that resulted from the training seemed to involve a freeing of the timing of the *Landing* and *Weighting* degrees of freedom. Analogous to the increase in JROM found in previous studies, batters in this group used a wider range of timings for these swing phases post-training as compared to pre-training (see Figures 3–5). However, although the variability and range of these swing phases increased with practice, it is questionable whether they were “frozen” at the start of training in the true sense proposed by Bernstein (1967). From Figures 4, 5, it is clear that, even in the pre-test, batters were using different timings for the *Landing* and *Weighting* phases for the two different pitch types. In other words, these variables were used as degrees of freedom in coordinating the swing. This lack of evidence for true freezing is perhaps not surprising, however, given that it is a coordination strategy proposed to occur early in learning while the participants in the Gray (2017) study were not novices and all had over 8 years playing experience at the time of the study.

In looking more closely at how the different phases of the swing were coordinated, the present analysis provides evidence that the functional couplings between swing phases are developed and strengthened with practice. For the VE Adaptive group, the correlations between the *Landing/Landing-Swing* and *Weighting/Weighting-Swing* phases were significantly more negative in the post-test, indicating stronger coupling. It is also notable that the magnitudes of correlation in the post-test for the Gray (2017) data were highly similar to those reported by Katsumata (2007). In Katsumata’s study, correlation values were −0.99 (slow) and −0.96 (fast) for *Landing/Landing-Swing* and −0.85 (slow) and −0.83 (fast) for *Weighting/Weighting-Swing*. Comparable values for VE Adaptive group in the post-test were −0.95, −0.95, −0.71, and −0.84. These similarities are what might be expected given that the high school players in the Gray (2017) study were at a lower level pre-training as compared to the college players used by Katsumata (2007). Finally, the adjustment in *Landing-Swing* phase that occurred with training in the present study is consistent with the finding of Nakata et al. (2013) that skilled batters seem to prepare the initial part of the swing (e.g., *Stepping* and *Landing*) for fastballs then wait for slower pitches during the *Landing-Swing* phase.

Combining these changes in the correlations between phases with the change in the variability in phases gives an indication of how the coordination solution was altered with training. Specifically, prior to training, batters in the VE Adaptive group seemed to rely more heavily on adjustments in the duration of the swing to get the bat to the hitting zone at the right time for the different pitch types. In other words, they swung faster for fastballs than changeups (Gray, 2002). This can be seen in Figure 3. The low variability in the timing of the bat reaching the hitting zone appears to be mostly produced by adjustments that occur after the onset of the *Swing*. Following training, batters in the present study seemed to rely more on online adjustments of the timing of their backwards and forwards weight shifts (in addition to changes in swing duration) to adjust for the different times of arrival of the fastballs and changeups. For example, they held their weight on their back foot longer for slower pitches. This is reflected in both the increase in the magnitudes of the correlations between the different swing phases and the pattern of variability shown in Figure 3. In the post-test, the low variability of the timing of the bat reaching the hitting zone was produced by online adjustments throughout the swing as evidenced by the significant decrease in variability between each adjacent phase.

As discussed in detail above, one of the limitations of previous work in this area is the lack of a demonstration of a direct link between the freeing of degrees of freedom that comes with practice and improvements in performance outcomes. To address this issue, the present study followed the logic of a UCM analysis and partitioned the variability in the timing of the phases of the swing into good and bad variability components. As predicted, for the VE Adaptive group, there was a significant increase in good variability (swing changes within the temporal constraint for successfully batting) and significant decrease in bad variability (swing changes outside the temporal constraint) from pre–post training. Furthermore, across all groups in the study, there were significant correlations between these changes in good/bad variability and measures of performance outcomes from VE and real hitting tests, and batting statistics in a full season of league play. This provides direct evidence that the freeing of timing of the different swing phases was functional in terms of performance. Finally, and consistent with the findings of Katsumata (2007), these findings suggest that successful hitting does not involve producing the same, low-variability, pre-programmed swing for every pitch (i.e., producing an “ideal” swing every time). Instead, it involves developing functional variability to allow for a swing that takes advantage of the good variability in order to be adaptable to different pitches.

How did these changes in swing coordination depend on the nature of the training conditions? As predicted, the other two training groups (that did more traditional, low variability batting practice in either the VE or against a pitching machine) and the control group (that did no additional training) from the Gray (2017) study showed less evidence of the development of functional couplings/coordinative structures between the swing phases from pre–post training. This included using less online adjustments of the weight shifts, no significant changes in the correlations between swing phases for changeups, and no significant changes in good variability following training. Consistent with the results of Lee et al. (2014) discussed above, it is proposed that these group differences occurred because the higher variability in practice conditions (including larger ranges and more pitch-pitch variation in speed, location and type) promoted more exploration of coordination solutions and consequently more use of the available degrees of freedom (redundancy).

A final novel issue that was addressed in the present study was how the change in the coordination solution following training depended on the individual constraints of the performer. To examine this, an individual constraint (bat speed) previously shown to have a large effect on the ability to make adjustments in hitting (Scott and Gray, 2010) was used. It was predicted that batters with a lower bat speed would be less able to take advantage of the available degrees of freedom in timing of the different swing phases. For example, a batter with a lower bat speed presumably cannot delay the *Weighting* phase of the swing as much as a batter with a higher bat speed or they would not be able to get the bat in the hitting zone in time for faster pitches. Thus, it was predicted that there should be a positive relationship between bat speed and changes in good and bad variability from pre–post training. Interestingly, this was only partially supported as bat speed was significantly related to the increase in good variability but not to the reduction in bad variability.

There are some important limitations to this study that should be addressed in future research. First, in several of the analyses used, data from different players are combined (e.g., to calculate the correlations between swing phases). Recent evidence suggests that how the problem of movement coordination is solved can show large individual differences even when similar levels of performance outcomes are achieved (Pacheco and Newell, 2018). This was also observed qualitatively in the Gray (2017) study. For example, some batters seem to delay the *Weighting* phase of the swing for changeups by holding the front foot in the air while others did it by initially landing softly on their front foot. These differences are lost in the group-level analyses. A second limitation is of course that all of the data used in these analyses were from swings made in a VE. Although it has previously been shown that this batting VE has high external validity (Gray, 2002, 2004), it will be important for the changes in coordination patterns observed in the present analysis to be tested in real batting. This is particularly important given the use of GRFs. Given that there was no force of contact at the instant of bat-ball contact in the VE (only a vibration), it is possible that the pattern of coordination is different from real batting.

The present analysis has some important practical applications for training baseball batters and athletes in other sports. From the results of the present study, it can be seen that solving the problem of movement coordination in batting seems to involve the development of a pattern of timing in the *Landing-Weighting-Swing* sequence that it is adaptable to different pitch types (task constraints) and the batter’s own abilities (individual constraints). The fact that the VE Adaptive group from the Gray (2017) showed both stronger functional couplings in coordinating the swing and a higher level of transfer to real baseball suggests that adding more variability in conditions to traditional batting practice will result in improved acquisition of the skill. Second, the finding that the individual constraint of bat speed influenced the extent to which batters can take advantage of the redundant degrees of freedom suggests both that (i) this is something that should be assessed and taken into account when coaching an individual batter and (ii) there could be potential motor leaning gains through strength and conditioning exercises specifically designed to increase bat speed. Finally, the present analysis adds to the growing body of evidence that for complex perceptual-motor skills like baseball batting, there is not one optimal technique that should be the same on every execution of the action.

The present study provided evidence that, consistent with Bernstein (1967) hypothesis, skill acquisition in baseball involves the freeing and development of functional couplings between degrees of freedom in movement, in particular the timing of the different phases of the swing. The coordinative structure that is developed takes advantage of the redundancy in degrees of freedom and good variability to be more adaptable for different pitch types. Finally, consistent with the model proposed by Newell (1986), how the problem of movement coordination is solved during training depends on constraints faced by the performer that are determined both by the structure of practice and by their individual capabilities.

## Data Availability Statement

Publicly available datasets were analyzed in this study. This data can be found here: http://perceptionaction.com.

## Ethics Statement

The studies involving human participants were reviewed and approved by the Arizona State University Institutional Review Board. The participants provided their written informed consent to participate in this study.

## Author Contributions

The author confirms being the sole contributor of this work and has approved it for publication.

## Conflict of Interest

The author declares that the research was conducted in the absence of any commercial or financial relationships that could be construed as a potential conflict of interest.
